# A comprehensive analysis of sequence variants and putative disease-causing mutations in photoreceptor-specific nuclear receptor *NR2E3*

**Published:** 2009-10-24

**Authors:** Atsuhiro Kanda, Anand Swaroop

**Affiliations:** Neurobiology Neurodegeneration and Repair Laboratory (N-NRL), National Eye Institute, National Institutes of Health, Bethesda, MD Departments of Ophthalmology and Visual Sciences and Human Genetics, University of Michigan, Ann Arbor, MI

## Abstract

**Purpose:**

The photoreceptor-specific orphan nuclear receptor NR2E3 is a key regulator of transcriptional events during photoreceptor differentiation in mammalian retina. Mutations in *NR2E3* are associated with enhanced S-cone syndrome and related retinal phenotypes that reveal characteristic excess of S-cone function. This study was undertaken to determine biochemical as well as functional consequences of reported sequence variants and disease-causing mutations in *NR2E3*.

**Methods:**

Twenty-five different mutations in the wild-type NR2E3 expression construct were generated by site-directed mutagenesis and performed nuclear localization, gel-shift, rhodopsin promoter activity assays, and co-immunoprecipitation in cultured mammalian cells.

**Results:**

Of the 25 mutant proteins, 15 mislocalize at least partially to the cytoplasm. Eight of the nine changes in the DNA-binding domain (DBD) and 12 of the 14 mutations in the ligand-binding domain (LBD) of NR2E3 exhibited reduced DNA-binding and transcriptional activation of the rhodopsin promoter. Moreover, these mutations dramatically altered the interaction of NR2E3 with NRL as well as with CRX. Two NR2E3 variants between DBD and LBD showed no effect on any biochemical or functional parameter tested.

**Conclusions:**

These data provide a better understanding of sequence variants, validate disease-causing mutations, and demonstrate the significance of DBD and LBD in mediating NR2E3 function. These studies contribute to molecular mechanisms underlying retinal phenotypes caused by NR2E3 mutations.

## Introduction

Retinal diseases represent a major cause of untreatable blindness in developed countries. Despite extensive phenotypic and genetic heterogeneity, these disorders primarily affect photoreceptor function [1-3] (RetNet website). Enhanced S-cone syndrome (ESCS; OMIM 268100) is a rare and somewhat unusual form of autosomal recessive retinal disease; the clinical characteristics include night blindness, an abnormal electroretinogram with a waveform that is nearly identical under both light and dark adaptation, an increased sensitivity of the electroretinogram to short-wavelength light, cystoid maculopathy, and degenerative changes of the vascular arcades [4-8]. The altered ratio of S- to L/M-cone photoreceptor sensitivity in ESCS was suggested to be due to abnormal cone cell fate determination during retinal development [8]. ESCS is caused by mutations in a photoreceptor-specific nuclear receptor nuclear receptor subfamily 2 group E member 3 (*NR2E3*, also called PNR; OMIM 604485) [9].

*NR2E3* was initially identified through a search of genes related to *NR2E1* (also called TLX or tailless) [10] and shown to be expressed in retinal photoreceptors [9,11,12]. A large number of sequence variants as well as possible mutations in *NR2E3* have been reported in patients with ESCS and other retinal phenotypes, which include autosomal dominant retinitis pigmentosa (ADRP), autosomal recessive RP (ARRP), Bardet-Biedl syndrome (BBS), clumped pigmentary retinal degeneration (CPRD), cone-rod dystrophy (CORD), and Goldmann-Favre syndrome (GFS) [9,13-24]. Consistent with the human phenotypes, a loss of function mutation in *Nr2e3*, caused by a L1 insertion, leads to increased S-cone function, slow degeneration of photoreceptors, and abnormal lamination of the outer nuclear layer with rosette formation in the retinal degeneration 7 (*rd7*) mouse [25,26]. While NR2E3 is suggested to be involved in controlling cone proliferation [27,28], overwhelming evidence has established NR2E3 primarily as a transcriptional suppressor of cone genes [12,29-32]. Moreover, NR2E3 is downstream of the rod differentiation factor neural retina leucine zipper (NRL) in transcriptional regulatory hierarchy [33] and can activate rod-specific genes (such as rhodopsin) synergistically with NRL or cone-rod homeobox (CRX) [12].

**Figure 1:**
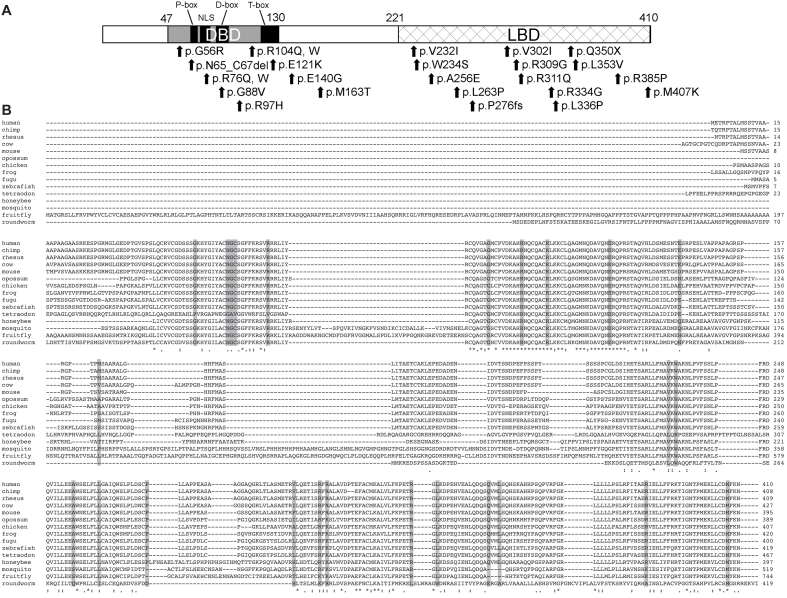
A schematic diagram of the human NR2E3 protein. **A**: Arrows indicate altered NR2E3 residues. Abbreviations: DNA binding domain (DBD), ligand binding domain (LBD), nuclear localization signal (NLS; NM_014249.2). **B**: NR2E3 protein sequence aligned to orthologs. By using ClustalW, we were able to align the sequence of human NR2E3 protein with those of chimp, rhesus, cow, mouse, opossum, chicken, frog, fugu, zebrafish, tetraodon, honeybee, mosquito, fruitfly, and roundworm. Amino acid residues conserved in all orthologs are indicated by asterisk, and lower identity or similarity is shown using either colon or a dot. Shadows indicate NR2E3 mutations analyzed in this report.

The analysis of NR2E3 primary sequence reveals two distinct regions: DNA-binding domain (DBD) close to the N-terminus, and ligand-binding domain (LBD) at the C-terminus [10]; a vast majority of human *NR2E3* variants/mutations are detected in these two domains (Figure 1A). Though the effect of a few has been evaluated [31,34,35], a comprehensive analysis of many variants and mutations has not been reported. This investigation was undertaken to distinguish *NR2E3* variants and polymorphisms from disease-causing mutations based on biochemical and functional parameters. Here, we describe the impact of 25 distinct sequence changes on NR2E3 protein localization, DNA-binding activity, transcriptional activation of rhodopsin promoter, and interaction with NRL and CRX.

## Methods

### Cell culture and transfection studies

COS-1 and HEK293 cells purchased from ATCC were cultured in Dulbecco’s modified Eagle’s medium containing 10% fetal bovine serum. Transient transfections of plasmid DNA were performed using FuGENE 6 (Roche Applied Science, Indianapolis, IN) at 80% cell confluency.

### Plasmid constructs and mutagenesis

The wild-type (WT) human *NR2E3* cDNA (GenBank NM_014249.2) was subcloned at EcoRI–NotI sites in the pcDNA4 His/Max C vector (Invitrogen, Carlsbad, CA) [12]. All mutants were generated by QuickChange XL site-directed mutagenesis kit (Stratagene, La Jolla, CA). The V5 epitope tag sequence was added at C-terminus of the pED-NRL and -CRX constructs [36] by site-direct mutagenesis. The pED expression vector is a derivative of pMT3 (Genetics Institute, Cambridge, MA). All constructs were sequence-verified before use.

### Immunoblotting and immunocytochemistry

COS-1 cells harbor large T-antigen and are suitable for transfection by vectors requiring expression of SV40 T-antigen. This allows expression of higher amount of protein for immunoblot analysis, immunoprecipitation, and immunocytochemistry. COS-1 cell extracts were solubilized in SDS-lysis buffer (2% SDS, 10% Glycerol, 0.062M Tris-HCl pH 6.8, 0.005% Bromophenol Blue) by heating to 100 °C for 5 min. Twenty μg proteins were separated by 12% SDS–PAGE and transferred to nitrocellulose by electroblotting. Immunoblot analysis was performed using a mouse monoclonal anti-Xpress antibody (Invitrogen), and immunostaining was performed essentially as described [37]. Briefly, COS-1 cells were transfected with plasmid DNA expressing WT or mutant NR2E3 protein. The cells were incubated with anti-Xpress antibody, followed by a secondary anti-mouse IgG Alexa fluor 488 (Invitrogen). Nuclei were counterstained with bisbenzimide.

### Electrophoretic mobility shift assay

Gel shift assays (EMSAs) were performed as described [10,29]. Briefly, the expression of the mutant NR2E3 protein in transfected COS-1 cell extracts was normalized by immunoblot analysis. The labeled probe DNA was initially incubated for 30 min on ice in binding buffer containing 10 mM Tris (pH 8.0), 1 mM DTT, 0.1% Nonidet P-40, 7.5% glycerol, and 0.1 mg/ml of poly(dI-dC). The Kni x2 oligonucleotides were Fw 5′-AGC TTA ACC CTT TTA AAA GTC AAA AGT CAA CTT CCA ACA GCT-3′; and Rv 5′-AGC TGT TGG AAG TTG ACT TTT GAC TTT TAA AAG GGT TAA GCT-3′. The nonspecific oligonucleotides were Fw 5′-AGC TTA ACC CTT TTA AAA TTC AAA ATT CAA CTT CCA ACA GCT-3′; and Rv 5′-AGC TGT TGG AAG TTG AAT TTT GAA TTT TAA AAG GGT TAA GCT-3′. Kni is derived from a TLL/TLX binding site found in the upstream region of *Drosophila melanogaster* knirps gene [10]. For competition experiments, nonradiolabeled oligonucleotides were used in 50 fold molar excess of the labeled oligonucleotides. Samples were loaded on a 5% nondenaturing polyacrylamide gel. After electrophoresis, gels were dried and exposed to X-ray film.

### Luciferase assays

HEK293 cells were used for luciferase reporter assays to allow relatively lower protein expression, thereby assisting in the evaluation of differences in transcriptional activities of mutant proteins. Luciferase reporter assays were performed using HEK293 cells, pGL2 with the bovine rhodopsin promoter driving a luciferase cDNA (BR130-luc), and expression vectors containing WT-CRX cDNA (pcDNA4-CRX) and NRL cDNA (pcDNA-NRL), as described [37]. The plasmid DNA expressing WT or mutant NR2E3 protein was cotransfected with pBR130-luc and pcDNA4-CRX as well as pcDNA4-NRL. Empty pcDNA4 expression vector and CMV-β-galactosidase plasmids were also included to normalize for the amount of transfected DNA and transfection efficiency, respectively. All transfections were performed in triplicate and repeated at least three times.

### Immunoprecipitation

WT or variant/mutant NR2E3 plasmid constructs were cotransfected into COS-1 cells with pED-NRL-V5 or -CRX-V5. The cells were harvested in 1× PBS containing protease inhibitors (Roche Applied Science). After an initial incubation of sonicated cell extracts with Protein-G beads (Invitrogen), goat anti-V5 antibody (Applied Biologic Materials Inc., BC, Canada) was added and left overnight at 4 °C with gentle mixing. The beads were washed with 1× PBS containing 1% Triton X-100, suspended in SDS sample buffer, and analyzed by SDS–PAGE.

## Results

### Human NR2E3 variants and mutations

To date, almost 50 *NR2E3* sequence changes (possible disease-causing mutations or polymorphic variations) have been reported in patients with ESCS, ADRP, ARRP, BBS, CPRD, CORD, and GFS [9,13-23]; of these, we have evaluated the following in this report: 22 missense (p.G56R [c.166G>A], p.R76Q [c.225G>A], p.R76W [c.226C>T], p.G88V [c.263G>T], p.R97H [c.290G>A], p.R104Q [c.311G>A], p.R104W [c.310C>T], p.E121K [c.361G>A], p.E140G [c.419G>A], p.M163T [c.488T>C], p.V232I [c.694G>A], p.W234S [c.701G>C], p.A256E [c.767C>A], p.L263P [c.788T>C], p.V302I [c.904G>A], p.R309G [c.925C>G], p.R311Q [c.932G>A], p.R334G [c.1000C>G], p.L336P [c.1007T>C], p.L353V [c.1057G>A], p.R385P [c.1154G>C] and p.M407K [c.1220T>A]) mutations, one nonsense (p.Q350X [c.1048C>T]), one deletion (p.N65_C67del [c.194_202del9]), and one frameshift (p.P276fs [c.827_843del17]) change (Figure 1A). The NR2E3 sequence is highly conserved during evolution (Ensemble). Residues G88, R97, R104, E121, and M407 are conserved in all NR2E3 orthologs from human to roundworm, whereas G56, N65, G66, C67, R76, and W234 are detected in all except tetradon (Figure 1B).

### Effect of NR2E3 mutations on subcellular localization

**Figure 2:**
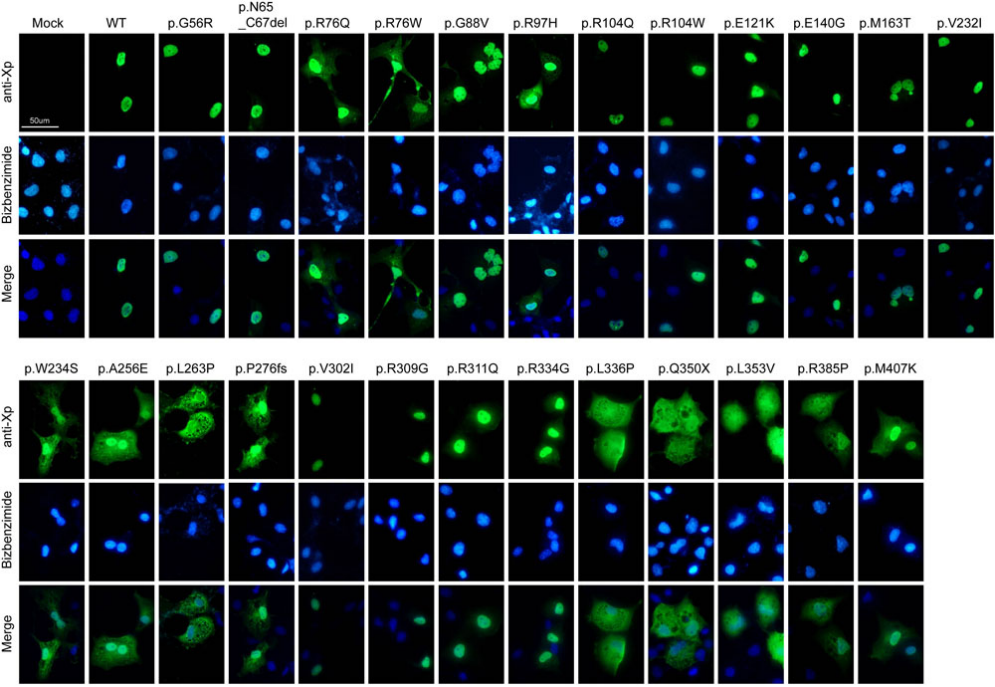
Subcellular localization of WT and mutant NR2E3 proteins in COS-1 cells. COS-1 cells expressing WT or mutant NR2E3 proteins were incubated with anti-Xpress antibody (anti-Xp) and visualized using anti-mouse IgG-Alexa 488 antibody (green). Central panels show nuclei labeled with bisbenzimide (blue). The bottom panels display merged images. Scale bar represents 50 μm.

We generated aforementioned 25 mutations in WT-NR2E3 expression construct and expressed the WT and mutant proteins in COS-1 cells. All mutant proteins displayed a predicted band size (45 kDa + 4 kDa Xpress epitope) upon immunoblot analysis, except two—p.P276fs and p.Q350X—because of premature truncation (data not shown). The WT-NR2E3 protein was detected in the nucleus of transfected COS-1 cells (Figure 2), consistent with nuclear localization of NR2E3 in rod photoreceptors of human and mouse retina [11,12]. Most mutations in NR2E3 (p.R76Q, p.R76W, p.R97H, p.W234S, p.A256E, p.L263P, p.P276fs, p.R309G, p.R311Q, p.R334G, p.L336P, p.Q350X, p.L353V, p.R385P, and p.M407K) resulted in partial or complete mislocalization of the expressed protein to the cytoplasm (Figure 2 and Table 1).

**Table 1:** Subcellular localization of mutant NR2E3 proteins.

**Amino acid change**	**Nuclear (%)**	**Nuclear and cytoplasmic (%)**	**Cytoplasmic (%)**
WT	97	2.7	0.3
p.G56R	95.3	3.7	1
p.N65_C67del	89.3	9	1.7
p.R76Q	56.7	29	14.3
p.R76W	52.5	26.8	20.7
p.G88V	90.5	6.3	3.2
p.R97H	59.5	29.2	11.3
p.R104Q	90.8	8	1.2
p.R104W	90.5	6	3.5
p.E121K	94.5	5	0.5
p.E140G	92.5	5.8	1.7
p.M163T	95.2	3.5	1.3
p.V232I	94.5	4.8	0.7
p.W234S	29.5	54.7	15.8
p.A256E	28.5	63.2	8.3
p.L263P	27.2	60.3	12.5
p.P276fs	13.5	79.7	6.8
p.V302I	92	6.2	1.8
p.R309G	83.5	15	1.5
p.R311Q	70.3	25.2	4.5
p.R334G	71.2	22.8	6
p.L336P	14.5	72	13.5
p.Q350X	5.5	80	14.5
p.L353V	5.5	74.2	20.3
p.R385P	35.2	58	6.8
p.M407K	38	59	3

Cellular localization of WT and mutant NR2E3 proteins was examined in COS-1 cells after transfection with the expression construct. Mutations affecting *NR2E3* nuclear localization (p.R76Q, p.R76W, p.R97H, p.W234S, p.A256E, p.L263P, p.P276fs, p.R309G, p.R311Q, p.R334G, p.L336P, p.Q350X, p.L353V, p.R385P, and p.M407K) demonstrate increased cytoplasmic staining. More than 200 cells were scored from at least two independent immunocytochemistry experiments.

### Effect of NR2E3 mutations on DNA-binding

**Figure 3:**
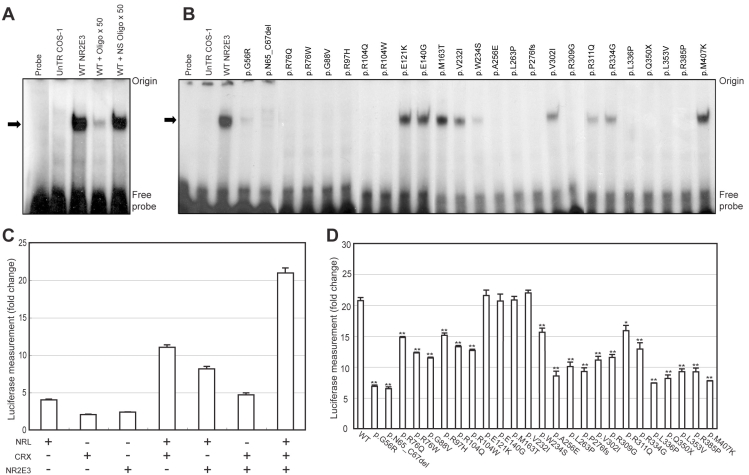
Effect of *NR2E3* mutations on DNA binding and transactivation. **A**: EMSAs were performed using the [^32^P]-labeled Kni x2 probe with untransfected (UnTR) COS-1 cells or WT *NR2E3* expressing cell extracts. Specificity of DNA binding is demonstrated by competition with unlabeled Kni x2 oligonucleotide (50×) and nonspecific (NS) oligonucleotide (50×). The arrow indicates the position of a specific DNA–protein complex between NR2E3 and Kni x2 oligonucleotide. **B**: Binding of mutant NR2E3 proteins to the labeled Kni x2 oligonucleotide was examined by EMSA. Mutant NR2E3 protein amount in cell extracts was normalized to WT-NR2E3 by immunoblot analysis. **C**: NR2E3 expression constructs were cotransfected into HEK293 cells with bovine rhodopsin-130 luciferase reporter plasmid and with NRL and CRX expression constructs. Fold change is relative to the empty expression vector control. Error bars indicate standard error of mean (SE). **D**: Luciferase assays were performed after co-transfection of mutant NR2E3 construct with the NRL and/or CRX expression constructs. ANOVA with a post hoc test were performed on each sample compared to WT NR2E3. Significant differences of p<0.05, and p<0.01 are shown as * and **, respectively. Error bars correspond to SEM.

The target sequence for binding of NR2E3 has not been described; however, a synthetic DNA including two AAGTCA half-sites separated by one spacer nucleotide (Kni x2) could bind to NR2E3 [10]. Kni is derived from a TLL/TLX binding site found in the upstream region of *Drosophila melanogaster* knirps gene [10]. Extracts from NR2E3-transfected COS-1 cells but not from untransfected or mock-transfected cells could bind to Kni x2 probe in EMSAs (Figure 3A). The intensity of shifted band was reduced by an excess of unlabeled Kni x2 oligonucleotide, but not nonspecific oligonucleotide (Figure 3A), as reported previously [10,29]. EMSA with p.V232I, p.W234S, p.V302I, p.R311Q, and p.R334G mutant proteins exhibit reduced binding to Kni x2 oligonucleotide compared to the WT. Several other mutants (p.N65_C67del, p.R76Q, p.R76W, p.R97H, p.R104Q, p.R104W, p.A256E, p.L263P, p.P276fs, p.R309G, p.L336P, p.Q350X, p.L353V, and p.R385P) showed no specific DNA binding (Figure 3B), even with a 10-fold molar excess of the transfected cell protein (data not shown). A faint signal was observed with p.G56R mutant protein in contrast to a recent study [35], probably because of different DNA binding conditions as well as cell lines used. Another report had revealed similar results with some of the mutants we studied (p.W234S, p.R311Q, p.385P, and p.407K) [34].

The NR2E3 protein expressed in COS-1 cells did not show binding to a predicted NR2E3 binding sites in the bovine rhodopsin promoter (data not shown), as these sequences (GAG CCA CGA GTC G or GCC TCA GAA GCA T) are somewhat different from the consensus (AAG TCA NAA GTC A) used in EMSA. It is possible that NR2E3 binding to rhodopsin promoter is mediated via its interaction with NRL or CRX [12].

### Effect of mutations on transcriptional activity of NR2E3

We used luciferase reporter activity assays to examine whether mutations affect the transcriptional regulatory activity of NR2E3 on bovine rhodopsin promoter. As reported earlier [12], NR2E3 synergistically activates the rhodopsin promoter through its interaction with NRL and CRX (Figure 3C). Mutations in the DBD (p.G56R, p.N65_C67del, p.R76Q, p.R76W, p.G88V, p.R97H, p.R104Q, and p.R104W) and LBD (p.A256E, p.L263P, p.P276fs, p.V302I, p.R309G, p.R311Q, p.R334G, p.L336P, p.Q350X, p.L353V, p.R385P, and p.M407K) demonstrated a variable reduction in NR2E3-mediated increase in transcriptional activity when mutant NR2E3 construct was cotransfected with both NRL and CRX (Figure 3D). Similar results were obtained when either NRL or CRX was used with mutant NR2E3 (data not shown). The data using R76W, R97H, and M407K mutants were consistent with previous studies [29,31]. The p.W234S and p.R311Q mutants showed different results from those reported [13,31], probably due to the differences in cell lines used as well as the length of bovine rhodopsin promoter.

### Effect of mutations and variants on the interaction of NR2E3 with NRL and CRX

**Figure 4:**
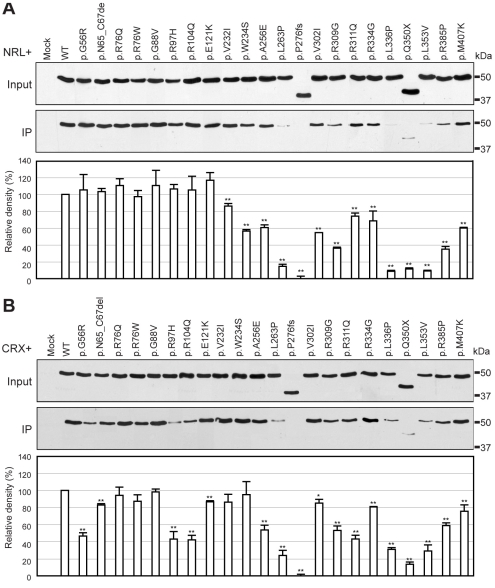
Interaction of WT and mutant NR2E3 proteins with NRL or CRX. **A**: Xpress-tagged WT or mutant NR2E3 expression plasmids were cotransfected into COS-1 cells with V5-tagged NRL expression construct. After immunoprecipitation with goat anti-V5 antibody, NR2E3 protein was detected with mouse anti-Xpress antibody. **B**: NR2E3 expression constructs were cotransfected into COS-1 cells with V5-tagged CRX expression construct. NR2E3 was visualized with mouse anti-Xpress antibody. In lower panels, the intensity of the WT NR2E3 immunoreactive band (normalized to input NR2E3) was compared with mutant NR2E3 proteins. ANOVA with a post hoc test were performed on each sample compared to WT NR2E3. Significant differences of p<0.05, and p<0.01 are shown as * and **, respectively.

NR2E3 interacts directly with NRL and CRX [12,31]. We therefore used cotransfection and coimmunoprecipitation assays to further evaluate the effect of mutations in DBD and LBD on the ability of NR2E3 to interact with NRL or CRX. The variations and mutations residing between DBD and LBD did not show any change in various assays; hence this study focused only on mutations in DBD and LBD. Reduced interaction with NRL is observed with p.W234S, p.A256E, p.L263P, p.P276fs, p.V302I, p.R309G, p.R311G, p.R334G, p.L336P, p.Q350X, p.L353V, p.R385P, and p.M407K mutants (Figure 4A). The p.G56R, p.N65_C67del, p.R97H, p.R104Q, p.E121K, p.A256E, p.L263P, p.P276fs, p.V302I, p.R309G, p.R311G, p.R334G, p.L336P, p.Q350X, p.L353V, p.R385P, and p.M407K mutations in NR2E3 revealed decreased interaction with CRX (Figure 4B).

## Discussion

Mutations in over 150 genes are associated with inherited retinal dysfunction as well as degeneration [2] (RetNet website). These genes encode proteins with diverse cellular functions, including phototransduction, intracellular transport, and transcriptional regulation. NRL, CRX, and NR2E3 are key transcription factors that regulate photoreceptor differentiation, and mutations in these lead to retinopathies [9,36,38,39]. We undertook this study to distinguish bona fide disease-causing mutations in NR2E3 from polymorphic variants based on biochemical and functional parameters and to examine the structure-function relationship (see Table 2 for a comprehensive summary). We show that mutations in the DBD and LBD of the NR2E3 protein result in functional changes and are likely to be disease-causing.

**Table 2:** A summary of effects of variants on NR2E3 function

**Proband diagnosis**	Nucleotide change*	Amino acid change*	**Alleles**	**Reference**	**IB**	**Subcellular localization**	**Binding to Kni x2**	**Rho-p Luc with NRL+CRX)**	**Interaction (NRL/CRX)**	**Effect**
-	Wild type	-	-	-	+	Nuc	+	+	+/+	-
ADRP	c.166G>A	p.G56R	p.G56R/+	19	No change	Nuc	Down	Down	Similar/Down	Mut
ESCS/GFS	c.194_202del9	p.N65_C67del	c.IVS1–2A>C/p.N65_C67del	9	No change	Nuc	Down	Down	Similar/Down	Mut
ESCS	c.225G>A	p.R76Q	p.R76Q/+	9	No change	Nuc, Cyt	Down	Down	Similar/Similar	Mut
ESCS	c.226C>T	p.R76W	p.R76W/+	9	No change	Nuc, Cyt	Down	Down	Similar/Similar	Mut
ESCS	c.263G>T	p.G88V	p.G88V/p.G88V	16	No change	Nuc	Down	Down	Similar/Similar	Mut
ESCS	c.290G>A	p.R97H	p.R97H/p.R97H	9	No change	Nuc, Cyt	Down	Down	Similar/Down	Mut
ESCS	c.311G>A	p.R104Q	p.R104Q/p.R334G	17	No change	Nuc	Down	Down	Similar/Down	Mut
ESCS	c.310C>T	p.R104W	c.IVS1–2A>C/p.R104W	9	No change	Nuc	Down	Down	ND	Mut
ESCS	c.361G>A	p.E121K	p.E121K/+	9	No change	Nuc	Similar	Similar	Similar/Down	Unc
BBS/RP	c.419G>A	p.E140G	p.E140G/+	9	No change	Nuc	Similar	Similar	ND	Var
BBS/RP	c.488T>C	p.M163T	p.M163T/+	9	No change	Nuc	Similar	Similar	ND	Var
BBS/RP	c.694G>A	p.V232I	p.V232I/+	9	No change	Nuc	Down	Similar	Down/Similar	Var
ESCS	c.701G>C	p.W234S	p.W234S/+	9	No change	Nuc, Cyt	Down	Down	Down/Similar	Mut
CPRD	c.767C>A	p.A256E	c.IVS1–2A>C/p.A256E	14	No change	Nuc, Cyt	Down	Down	Down/Down	Mut
ESCS	c.788T>C	p.L263P	c.IVS1–2A>C/p.L263P	16	No change	Nuc, Cyt	Down	Down	Down/Down	Mut
CPRD	c.827_843del17	p.P276fs	c.IVS1–2A>C/p.P276fs	14	No change	Nuc, Cyt	Down	Down	Down/Down	Mut
BBS/RP	c.904G>A	p.V302I	p.V302I/+	9	No change	Nuc	Down	Down	Down/Down	Unc
ESCS	c.925C>G	p.R309G	p.R309G/p.R311Q	9	No change	Nuc, Cyt	Down	Down	Down/Down	Mut
ESCS	c.932G>A	p.R311Q	p.R311Q/p.R311Q	9	No change	Nuc, Cyt	Down	Down	Down/Down	Mut
ESCS	c.1000C>G	p.R334G	p.R104Q/p.R334G	17	No change	Nuc, Cyt	Down	Down	Down/Down	Mut
ESCS	c.1007T>C	p.L336P	c.IVS1–2A>C/p.L336P	16	No change	Nuc, Cyt	Down	Down	Down/Down	Mut
ESCS	c.1048C>T	p.Q350X	p.Q350X/p.Q350X	15	No change	Nuc, Cyt	Down	Down	Down/Down	Mut
ESCS	c.1057G>A	p.L353V	c.481delA/p.L353V	16	No change	Nuc, Cyt	Down	Down	Down/Down	Mut
ESCS	c.1154G>C	p.R385P	p.R385P/+	9	No change	Nuc, Cyt	Down	Down	Down/Down	Mut
ESCS	c.1220T>A	p.M407K	p.M407K/p.M407K	9	No change	Nuc, Cyt	Similar	Down	Down/Down	Mut

The results of nuclear localization, EMSA, luciferase assays and immunoprecipitations for each mutation are summarized in this table. Each alteration is categorized as a mutation, variation or change of uncertain significance based on the experiments reported in this study. Abbreviations: retinitis pigmentosa (RP), autosomal dominant RP (ADRP), Bardet-Biedl syndrome (BBS), clumped pigmentary retinal dystrophy (CPRD), enhanced S-cone syndrome (ESCS), Goldmann-Favre syndrome (GFS), mutation (Mut), no data (ND), cytoplasm (Cyt), nuclear (Nuc), uncertain significance (Unc), variation (Var), rhodopsin promoter driving luciferase (Rho-p Luc). *The mutation nomenclature uses the first nucleotide of the ATG codon in the cDNA (NM_014249.2) as +1, and the first codon of the protein as +1 (NM_014249.2).

In NR2E3-DBD, a proximal box (P-box; residues 65 to 69) and a distal box (D-box; residues 84 and 90) bind to the half-site core sequence (AAGTCA) and are critical for determining DNA-binding specificity and dimerization, whereas the T-box region (residues 114 to 130) makes a helix and correlates to a dimerization surface [10,40]. Although Coppieters et al. [19] suggested a putative nuclear localization signal between residues 72 and 78 in NR2E3, our data show that a single amino acid change in the nuclear localization signal is not sufficient to change the cellular localization of NR2E3. The p.N65_C67del mutant lacks three residues (Asn-65, Gly-66, Cys-67) in the P-box. Mutations in highly conserved residues at position 56, 97, and 104 that are located within the zinc finger region involved in DNA binding [41] affect transcriptional activity. Interestingly, the changes at residues 56 and 104 reduced the interaction of NR2E3 with CRX but not NRL, probably because of altered protein interaction surfaces. These studies suggest that the nature of interactions between NR2E3 and NRL or CRX and the conformation of the DBD of NR2E3 could be critical for differential regulation of gene expression in photoreceptors.

Crystal structures of retinoid X receptor-α (RXRα) reveal that LBD in nuclear receptors consists of 12 α-helices (H1 to H12) [42,43]. The region from H3 to H4 contains a LBD-specific signature motif [(F/W)AKxxxFxxLxxxDQxxLL] that is involved in dimerization and transactivation and holds together H3–5, H8, and H9 [44,45]. Trp-234 is one of the residues constructing the signature motif, and as expected, p.W234S mutation leads to the loss of NR2E3 function, as in case of a similar estrogen receptor-α mutation [46]. Mutations in Val-302 and Arg-311 residues that are expected to participate in anchoring interactions between helices [42,47] appear to affect NR2E3 function in the assays we used. The dimerization interface is mainly located in H9 and H10 [42], suggesting that p.L353V and p.R385P mutants in these regions may impair dimerization. Met-407 is located within H12, which covers the ligand-binding cavity and stabilizes ligand binding by contributing to the hydrophobic pocket [43]. Substitution of Leu-263 (H5) and Leu-336 (loop 8–9) to Proline completely eliminated NR2E3 function, probably because of altered NR2E3 conformation [16]. The p.P276fs and p.Q350X mutations are completely missing the latter half part of the LBD, thereby resulting in a loss of function. Other mutations (p.A256E of H5, p.R309Q of H7, p.R334G of loop 8–9) decreased the ability of NR2E3 to interact with NRL and CRX. As co-activators are necessary for ligand-activated nuclear receptors to stimulate transcription [48], amino acid substitutions in NR2E3 helices or loops that selectively affect interaction with NRL as well as CRX are expected to alter transcriptional regulation of downstream genes. Most of the alterations in the LBD exhibited reduced DNA binding. This mutant fused to Gal4-DBD was previously reported to impair transcriptional suppression [29]. We hypothesize that certain LBD residues in NR2E3 contribute to unique structural motifs or participate in dimerization, and mutations at these sites may alter interactions resulting in weaker or diminished DNA binding. The significant role of LBD in NR2E3 functions implicates as a yet to be determined ligand that may control NR2E3 activity in rod photoreceptors.

The p.E121K mutation in the T-box within DBD and p.V232I change in LBD-H3, together with two variations detected between DBD and LBD (p.E140G and p.M163T), demonstrated no effect on biochemical as well as functional parameters that we have tested. Notably, p.E140G, p.M163T, and p.V232I variants were detected in BBS and RP patients as well as in control individuals [9]. The p.E121K mutation was identified in an ESCS patient and is located within the T-box [10]. These data reveal that p.E140G, p.M163T, and p.V232I are not likely to be disease-causing mutations; however, additional experiments are required to validate the association of p.E121K with retinal disease. It is possible that mutations including p.E121K affect interaction with other proteins (e.g., NR1D1) as well as the activity of other promoters (e.g., Pde6b, Gnat1, S-opsin). However, one should be cautious in interpreting the in vivo consequence of these mutants as the studies reported here were performed in vitro.

So far, five homozygous mutations (p.G88V, p.R97H, p.R311Q, p.Q350X, and p.M407K) and 15 compound heterozygotes (p.G56R, p.N65_C67del, p.R76Q, p.R76W, p.R104Q, p.R104W, p.W234S, p.A256E, p.L263P, p.P276fs, p.R309G, p.R334G, p.L336P, p.L353V, and p.R385P) have been reported in ESCS, ADRP, as well as CPRD probands. Clinical investigations, together with this study, confirm that all of these changes are disease-causing. In addition to 25 variants tested here, three additional missense mutations (p.V49M, p.Y81C, and p.A256V), three intron splice-acceptor mutations (IVS1–2A>C, IVS1–3C>G and IVS8–1G>A), and a one base-pair deletion at position 481 (c.481delA), have been reported in ESCS patients [9,16,22]. IVS1–2A is among the most common NR2E3 mutations, whereas 481delA causes a frameshift leading to 17 abnormal residues followed by a premature stop codon. The p.S44L, p.A63D, p.F71del, p.R77Q, p.G287S, p.K324R, p.K345X, and p.Q350R mutations have also been reported in unaffected controls and some retinopathy patients [18,19,23,24].

In conclusion, this study establishes that ESCS and related retinal phenotypes result from partial or complete loss of NR2E3 function in photoreceptors. Disease-causing NR2E3 mutations are associated with diminished transcriptional regulatory activity, which can be due to altered subcellular localization, reduced DNA binding or less-strong interaction with co-regulators (such as NRL and CRX). Both DBD and LBD are essential for NR2E3 function. The *rd7* mouse offers an excellent model to investigate the mechanism of disease caused by the loss of NR2E3 function [12,29]. We hypothesize that (i) a lack of or reduced NR2E3 activity in developing retina of retinopathy patients does not allow functional maturation of rod photoreceptors; (ii) enhanced S-cone function is the result of de-repression of S-cone genes in such photoreceptors; (iii) these abnormal rod photoreceptors (expressing cone genes) degenerate with time; and (iv) different clinical phenotypes may reflect distinct impact of mutations on the transcriptional regulatory function of NR2E3.
